# Supplementary material for: Translation is required for miRNA‐dependent decay of endogenous transcripts
Source: EMBO J. 2020 Dec 10;40(3):e104569. doi: 10.15252/embj.2020104569 (PMC7849302; doi:10.15252/embj.2020104569)
Supplement: Supplementary file 1 — Appendix [file EMBJ-40-e104569-s001.pdf]

Appendix: Table of contents

Appendix Figures and Table legends 2

Appendix Figure S1 3

Appendix Figure S2 4

Appendix Table S1 5

Appendix Table S2 6

Appendix Figure S1 – MiRNA binding sites found within *lncRNAc1* sequence are conserved. Alignment in mouse, rat and human of the mutated MRE sequences in *lncRNA-c1*. Seed region is highlighted in grey. = represent positions not conserved and – deletions in either rat or human. Deletions in mouse are represented in yellow and the length indicated in the alignment.

Appendix Figure S2 - Exemplar Flow Cytometry results for cells transfected with mock, GFP, BoxB(-30)GFP, GFP-lncRNAc1, BoxB(-30)GFP-lncRNAc1, GFP-lncRNAc2 and BoxB(-30)GFP-lncRNAc2. Each row, **(A)** represents side scatter intensity (SSC-A, y-axis) as a function of forward scatter intensity (FSC-A, x-axis). The percentage of gated events (cells) is shown on the lower left corner. **(B)** represents GFP intensity (x-axis) as a function of FL2 (auto-fluorescence, y-axis). **(C)** Percentage of GFP positive cells is shown on the lower right corner of the panel. Right panel represents the distribution of GFP fluorescence (x-axis) as a function of the number of cells (count, y-axis). Portion of this figure was used to assemble Figure 4D.

Appendix Table S1 – Reagents and Tools Table. All reagents and bioinformatic tools used in the study are listed with respective manufacturers and catalogue numbers.

Appendix Table S2 – Primer names and sequence table. All primers used in this study are listed with respective sequence and implementations.

Appendix Figure S1

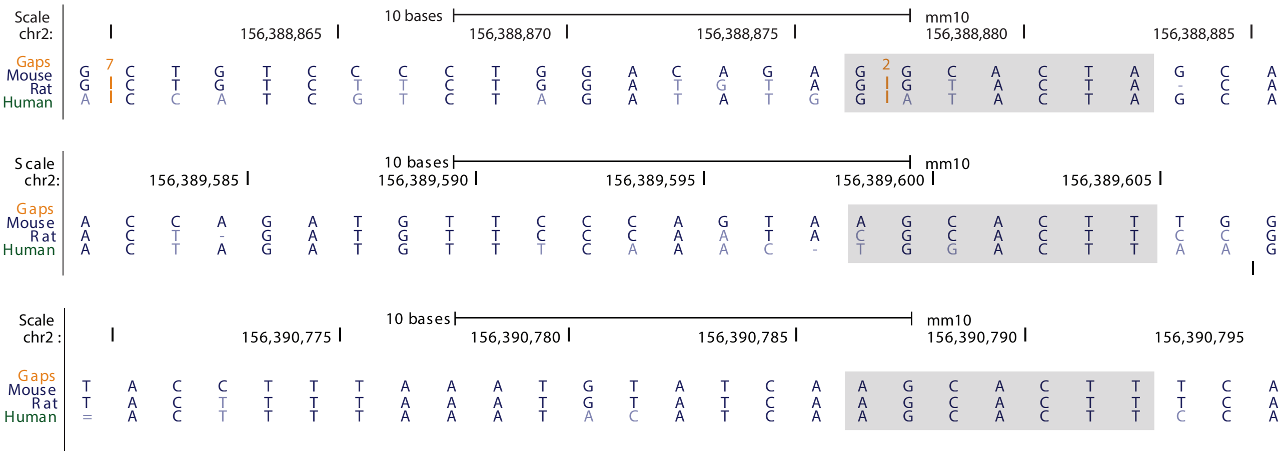

Appendix Figure S2

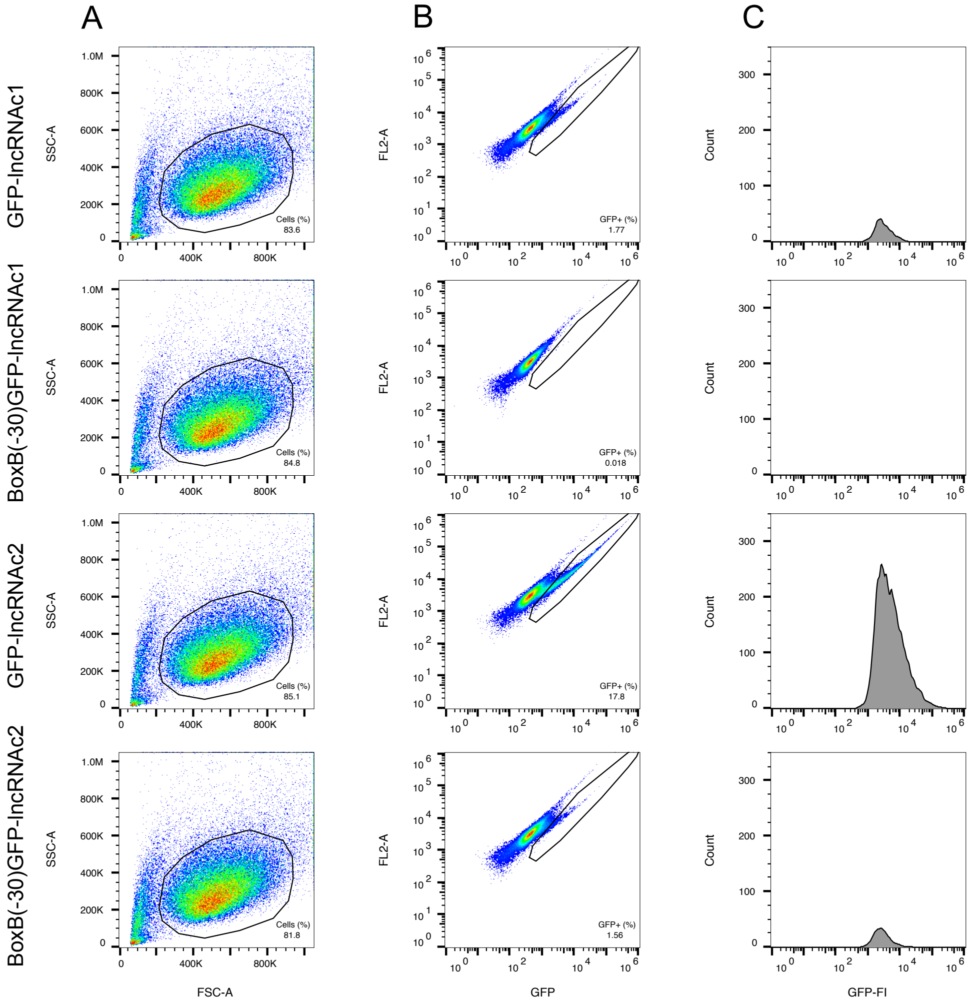

APPENDIX TABLE S1: Reagents and tools table		
**Reagent/Resourse**	**Reference or source**	**Identifier or catalog number**
**Antibodies**		
Rabbit anti-AGO2	Cell Signaling	#2897
normal rabbit IgGs	SIGMA	I5006
Rabbit anti-DICER	SIGMA,	SAB4200087
anti-Rabbit IgG-HRP	Cell Signaling,	#7074
Mouse anti-AGO2	FujiFilm Wako P.C. Corp.	018-22021
Rabbit anti-DCR	Santa Cruz Biotechnology	H-212: sc-30226
Rabbit anti-NANOG	Abcam	Ab70482
Rabbit anti-OCT4	Abcam	Ab27985
Mouse anti-ACTIN-β	SIGMA	A2228-100UL
Goat Anti-Rabbit IgG (H+L)	BIORAD	170-6515
Goat Anti-Mouse IgG (H+L)	BIORAD	170-6516
Rabbit Anti-Goat IgG/HRP	Dako (Agilent)	P0449
**Chemicals, Peptides, and Recombinant Proteins**		
[Z]-4-Hydroxytamoxifen	Sigma	H7904
β-mercaptoethanol	Thermo Fischer	31350-10
Recombinant mouse Leukemia Inhibitory factor	Merck	ESG1107
4sU	Sigma	T4509
biotin-HPDP	Thermo Fisher	21341
TURBO DNAse	Thermo Fisher	AM2238
Actinomycin D	Thermo Fisher	11805017
protease inhibitors	Roche	11697498001
RNase inhibitors	Thermo Fisher	EO0381
DNase	Promega	M6101
Tris Base	Applichem	A1379, 1000
NaCl	Applichem	A2942, 1000
Glycine	Applichem	A1067, 1000
SDS	Applichem	A2263, 0100
Methanol	SIGMA	32213,1L
Hydrochloric Acid fuming 37%	ROTH	4625.1
Phenol-chloroform-Isoamyl alcohol mixture	SIGMA	77618-500ML
Chloroform	SIGMA	C2432-500ML
Sodium-Azide	SIGMA	S2002
Ponceau S Solution	SIGMA	P7170
NheI-HF	NEB	R3131S
XhoI	NEB	R0146S
EcoRI-HF	NEB	R3101S
DpnI	NEB	R0176S
T4 DNA ligase	NEB	M0202S
**Critical Commercial Assays**		
DNAse on column digestion	Qiagen	74104
TruSeq small RNA Library Prep kit	Illumina	NA
Qiagen RNeasy Mini Kit	Qiagen	74104
Ovation RNA-Seq System V2	Tecan Genomics	7102-08
TruSeq Nano DNA Low Throughput Library Prep Kit	Illumina	20015964
PARIS kit	Thermo Fisher	AM1921
miRNeasy kit	Qiagen	217004
Quantitect Reverse Transcription Kit	Qiagen	205310
FastStart DNA Essential DNA Green Master	Roche	6924204001
Applied Biosystems Taqman microRNA Reverse Transcription Kit	Thermo Fisher	4366596
Taqman Universal Master Mix II	Thermo Fisher	4440043
GoScript RT Kit	Promega	A5004
SuperSignalTM West Femto Maximum Sensitivity Assay	Thermo Fisher	34095
Infusion HD Cloning kit	Takara	121416
PierceTM BCA Protein Assay Kit	Thermo Fisher	23225
**Deposited Data**		
mESC small RNA seq	This paper	GEO: GSE143277
mESC 4sU-seq	This paper	GEO: GSE143277
mESC HEAP	Li X., et al, 2020	GEO: GSE13934
mESC AGO2-CLIP	Leug, KLA, et al, 2011	GEO: GSE25310
mESC Ribosomal Profiling	Ingolia, N et al, 2011	GEO: GSE30839
**Experimental Models: Cell Lines**		
Mouse DTCM23/49 XY embryonic stem cells	Graham B. et al, 2016	N/A
**Oligonucleotides**		
Primers sequences, see Table EV3	This paper	N/A
mmu-miRNA294-3p inhibitors	Thermo Fisher	MH10865
mmu-miR294-3p mimics	Thermo Fisher	MC10865
mmu-miR295-3p mimics	Thermo Fisher	MC10386
miRNA mimic negative controls	Thermo Fisher	4464059
mmu-miR-290-3p Taqman probe	Thermo Fisher	2591
mmu-miR-295-3p Taqman probe	Thermo Fisher	189
snoRNA202	Thermo Fisher	1232
**Recombinant DNA**		
pcDNA3.1(-) plasmid	Addgene	V79520
**Software and Algorithms**		
Cutadapt	Martin, M, 2011	DOI:10.14806/ej.17.1.200.
STAR	Dobin, A et al, 2013	https://github.com/alexdobin/STAR
RSEM	Bo, L, et al, 2011	http://deweylab.biostat.wisc.edu/rsem
INSPEcT	De Pretis, S et al, 2015	https://bioconductor.org/packages/release/bioc/html/INSPEcT.html
Bowtie	Langmead, B, et al, 2009	http://bowtie-bio.sourceforge.net/index.shtml
PARalyzer	Corcoran, D et al, 2011	https://ohlerlab.mdc-berlin.de/software/PARalyzer_85/
BEDtools	Quinlan, AR,et al, 2010	https://bedtools.readthedocs.io/en/latest/
Bowtie 2	Langmead B et al, 2012	http://bowtie-bio.sourceforge.net/bowtie2/index.shtml
edgeR	Robinson, M et al, 2010	https://bioconductor.org/packages/release/bioc/html/edgeR.html
**Other**		
DMEM culture medium	Thermo Fisher	41965-039
100 X Non-Essential Amino Acids	Thermo Fisher	11140-035
Fetal Bovine Serum	Thermo Fisher	10499-044
Penicillin/Streptomycin	Thermo Fisher	15140122
Trizol	Thermo Fisher	15596026
DynabeadsTM MyOneTM Streptavidin T1 beads	Thermo Fisher	65601
DynaMagTM-2 Magnetic stand	Thermo Fisher	12321D
lipofectamine 2000	Thermo Fisher	12566014
RNAimax transfection reagent	Thermo Fisher	13778150
Protein A/G Plus-Agarose beads	Santa Cruz Biotechnology	sc-2003
Qiagen 2 ml phase lock tubes	Qiagen	129056
Qiagen 15 ml phase lock tubes	Qiagen	129065
NuPageTM 12% Bis-Tris Gel	Thermo Fisher	NP0341BOX
Advansta ECL Western Bright	Advansta	K-12045-D20
10 X Cutsmart Buffer	NEB	B7204S
10 X T4 DNA ligase Buffer	NEB	B0202S
Propium Iodide Staining Solution	BD Biosciences	550825

APPENDIX TABLE S2 - Primer names and sequence table.

PrimerTarget	Primer Sequence (5' -> 3')	application
Actin-β_fwd	GGCTGTATTCCCCTCCATCG	qPCR analysis
Actin-β_rev	CCAGTTGGTAACAATGCCATGT	qPCR analysis
Ccne2_fwd	AACCCAGATAATTCAGGCCAAG	qPCR analysis
Ccne2_rev	CAGTACAGGTGGCCAACAATTC	qPCR analysis
Cdkn1a_fwd	CGAGAACGGTGGAACTTTGAC	qPCR analysis
Cdkn1a_rev	CAGGGCTCAGGTAGACCTTG	qPCR analysis
Dbt-201_fwd	TCACCATCACCAGCCGTTAC	qPCR analysis
Dbt-201_rev	GCGGGGGTTTCAACAACATC	qPCR analysis
Dicer_fwd	CGATATTCGATCCTCCTGT	qPCR analysis
Dicer_rev	GCCAGCAAGCAGTCTTTTGT	qPCR analysis
E4f1_fwd	GAGGCCACCTCTCTAGCAGAA	qPCR analysis
E4f1_rev	GCCATCTCGACATCCCCTG	qPCR analysis
EGFP_fwd	CTGAAGGGCATCGACTTCA	qPCR analysis
EGFP_rev	CTTGTCGGCCATGATATAGA	qPCR analysis
GapdH_fwd	CGTATTGGGCGCCTGGTCAC	qPCR analysis
GapdH_rev	ATGATGACCCTTTTGGCTCC	qPCR analysis
Lats2_fwd	GACAAGTCCATGTTTGTGAAAATCA	qPCR analysis
Lats2_rev	TTCCGGTTCAGGACATCCTT	qPCR analysis
lncRNA-c1_fwd	CAGCTGGGAGACACACCTAC	qPCR analysis
lncRNA-c1_rev	GTTGCTAGGCTGACTGGAGG	qPCR analysis
lncRNA-c2_fwd	GATGCCGCCACAAAGAGTTC	qPCR analysis
lncRNA-c2_rev	CCACTCATCTGACCACTCCG	qPCR analysis
Malat1_fwd	TAAGCGCTTGCCTCTGTCTT	qPCR analysis
Malat1_rev	CACCTGCATTCTGTGTGGTC	qPCR analysis
Myc_fwd	ATCAGCAACAACCGCAAGTG	qPCR analysis
Myc_rev	TCTGACGTTCCAAGACGTTGTG	qPCR analysis
Nanog_fwd	GAACTATTCTTGCTTACAAGGGTCTGC	qPCR analysis
Nanog_rev	GCATCTTCTGCTTCCTGGCA	qPCR analysis
Neomycin_fwd	CTTGGGTGGAGAGGCTATTC	qPCR analysis
Neomycin_rev	AGGTGAGATGACAGGAGATC	qPCR analysis
Oct-4_fwd	CGTGGAGACTTTGCAGCCTG	qPCR analysis
Oct-4_rev	GCTTGGCAAACTGTTCTAGCTCCT	qPCR analysis
PolII_fwd	GACGACGATGAGATCACTCCG	qPCR analysis
PolII_rev	GGTGCATCTTCCACAATTCTTTG	qPCR analysis
Rbl2_fwd	ACTATGGAACCTTGCTCCTGAATC	qPCR analysis
Rbl2_rev	CCCCTACCCCTTGGGTGAT	qPCR analysis
Sox2_fwd	TGGAAACTTTTGTCCGAGA	qPCR analysis
Sox2_rev	GAAGCGTGTACTTATCCTTCTTCAT	qPCR analysis
TCONS00031378_f	GATGCCGCCACAAAGAGTTC	qPCR analysis
TCONS00031378_r	CCACTCATCTGACCACTCCG	qPCR analysis
GFP_cloning_fwd	AGCATGCTGCTAGCATGGTGAGCAAGGGCGAGGA	amplification of EGFP and insertion into into pcDNA3.1(-)
GFP_cloning_fwd	AGCATGCTGCTAGCTTACTTGTACAGCTCGTCCA	amplification of EGFP and insertion into into pcDNA3.1(-)
lncRNAc1_cloning_fwd	AGCATGCTCTCGAGGTCACCCAGTCACAAGCAGC	amplification of candidate lncRNAc1 from cDNA and insertion into pcDNA3.1(-) through XhoI and EcoRI directional cloning
lncRNAc1_cloning_rev	AGCATGCTGAATTCCAAACCTCTCCTGAGTTGAC	amplification of candidate lncRNAc1 from cDNA and insertion into pcDNA3.1(-) through XhoI and EcoRI directional cloning
lncRNAc1_MRE1fwd	CAGTATTCACGATGGTTTCCCCAGCATGTG	scrambling of lncRNAc1 MRE1 (Infusion)
lncRNAc1_MRE1rev	CCATCGTGAATACTGGGAACATCTGGTCTCCAG	scrambling of lncRNAc1 MRE1 (Infusion)
lncRNAc1_MRE2fwd	AAATGTATCATTCACGATCAGATATCTAAAAGACTAGATGTTTCATG	scrambling of lncRNAc1 MRE2 (Phusion HF)
lncRNAc1_MRE2rev	AGATATCTGATCGTGAATGATACATTTAAAGGTACAGGTAATAGAAT	scrambling of lncRNAc1 MRE2 (Phusion HF)
lncRNAc1_MRE3fwd	ACAGAATCACGGGCAGCCTTTCCCTCAGCCA	scrambling of lncRNAc1 MRE3 (Infusion)
lncRNAc1_MRE3rev	TGCCCGTGATTCTGTCCAGGGGACAGCTCT	scrambling of lncRNAc1 MRE3 (Infusion)
lncRNAc2_cloning_fwd	AGCATGCTCTCGAGCAGGAATAGAAACCCTGACT	amplification of candidate lncRNAc2 from cDNA and insertion into pcDNA3.1(-) through XhoI and EcoRI directional cloning
lncRNAc2_cloning_rev	AGCATGCTGAATTCACGGACTCTCAGTTCCTCTT	amplification of candidate lncRNAc2 from cDNA and insertion into pcDNA3.1(-) through XhoI and EcoRI directional cloning
Cnstruct_Amp_(-30bp)_fw	ATGGTGAGCAAGGGCGAGG	PCR amplification of GFP/GFP-lncRNA-c1/c2 construct to inser BoxB hairpins -30 bp upstream of the coding region of GFP
Cnstruct_Amp_(-30bp)_rev	GCTAGCCAGCTTGGGTCTCC	PCR amplification of GFP/GFP-lncRNA-c1/c2 construct to inser BoxB hairpins -30 bp upstream of the coding region of GFP
BoxB_Insert_(-30)_fw	CCCAAGCTGGCTAGCCACAAAGCCGCTCCATCAGC	PCR amplification of 5 BoxB hairpins from (pAc5.1C-5BoxB73-Fluc-STOP-CG10011-SV40) for insertion upstream of the coding region of GFP
BoxB_Insert_(-30)_rev	GCCCTTGCTCACCATATGGGTGACCTCGAGATAATATCC	PCR amplification of 5 BoxB hairpins from (pAc5.1C-5BoxB73-Fluc-STOP-CG10011-SV40) for insertion upstream of the coding region of GFP
Cnstruct_Amp_(+339bp)_fw	AGGACGACGGCAACTACAAGAC	PCR amplification of GFP/GFP-lncRNA-c1/c2 construct to inser BoxB hairpins +339 bp into the coding region of GFP
Cnstruct_Amp_(+339bp)_rev	TGAAGAAGATGGTGCGCTCCTG	PCR amplification of GFP/GFP-lncRNA-c1/c2 construct to inser BoxB hairpins +339 bp into the coding region of GFP
BoxB_Insert_(+339)_fw	GCACCATCTTCTTCACTAGCCACAAAGCCGCTCC	PCR amplification of 5 BoxB hairpins from (pAc5.1C-5BoxB73-Fluc-STOP-CG10011-SV40) for insertion in the middle of coding region
BoxB_Insert_(+339)_rev	AGTTGCCGTCGTCCATGGGTGACCTCGAGATAATATCC	PCR amplification of 5 BoxB hairpins from (pAc5.1C-5BoxB73-Fluc-STOP-CG10011-SV40) for insertion in the middle of coding region
