# Supplementary material for: Translation is required for miRNA‐dependent decay of endogenous transcripts
Source: EMBO J. 2020 Dec 10;40(3):e104569. doi: 10.15252/embj.2020104569 (PMC7849302; doi:10.15252/embj.2020104569)
Supplement: Supplementary file 2 — Expanded View Figures PDF [file EMBJ-40-e104569-s002.pdf]

Expanded View Figures

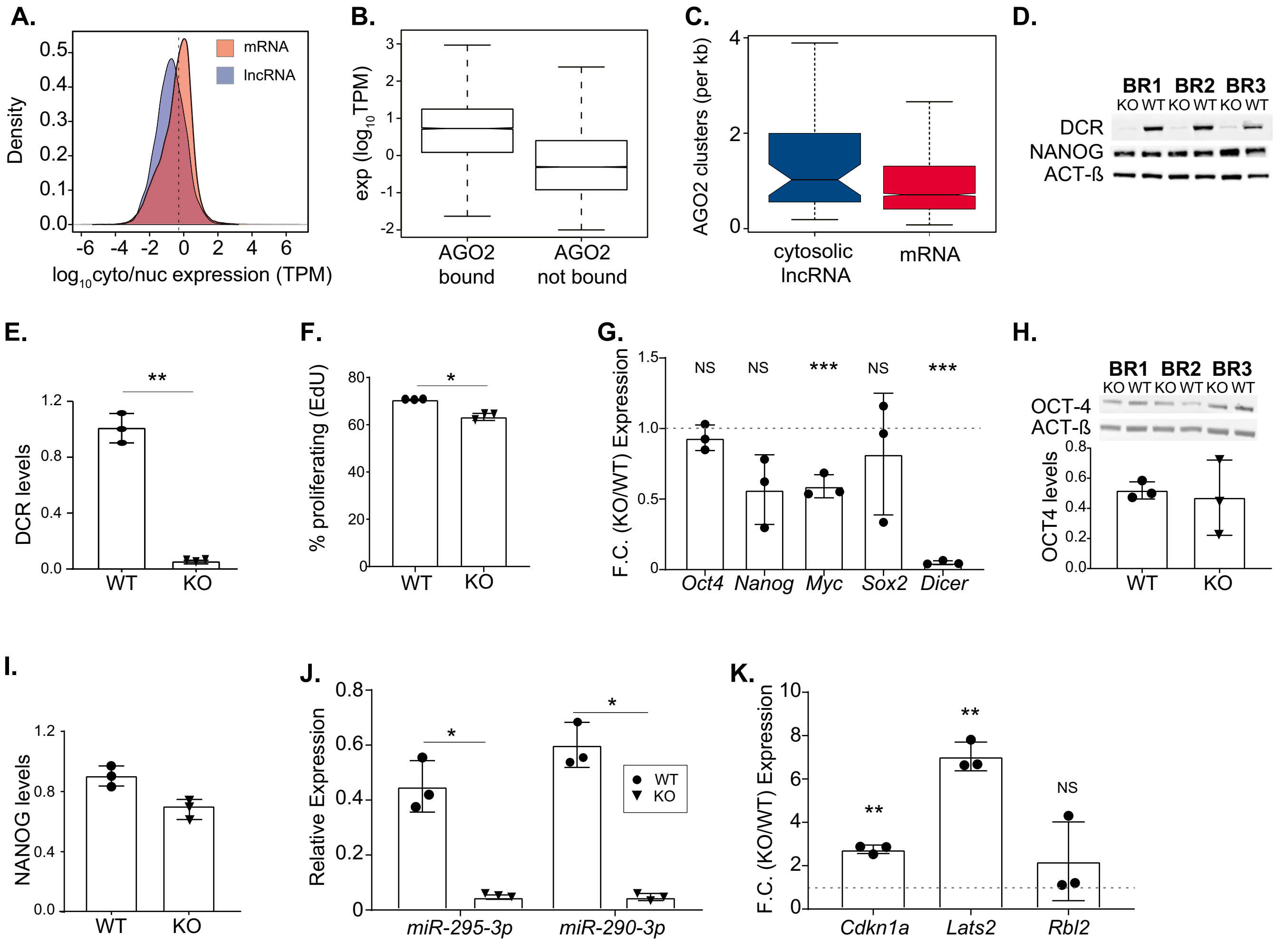

Figure EV1.

© 2021 The Authors

Figure EV1. Characterization of miRNA-depleted mESC following inducible *Dicer* knockout.

A Distribution of log_{10} ratio between nuclear/cytosolic (transcripts per million, TPM) in mESCs for mRNAs (red) and lncRNAs (blue). Median nuclear/cytosolic ratio of mRNAs is represented by dashed line.

B Distribution of the expression (log_{10}(TPM)) of transcripts with and without experimental evidence for AGO2 binding in mESCs. Central band of boxplot represents median, box depicts 25–75 quantiles of distribution, and whiskers represent the 5th and 95th quantiles of the distribution. The data represented are based on analysis of two independent biological replicates.

C Density of AGO2 wild-type specific clusters across cytosolic lncRNAs (n = 48, blue) and the 3'untranslated regions of mRNAs (n = 2,355, red) with experimental evidence for AGO2 binding in mESC (>0 AGO2 clusters) based on (Leung *et al*, 2011). Central band of boxplot represents median, box depicts 25–75 quantiles of distribution, and whiskers represent the 5th and 95th quantiles of the distribution.

D Immunoblot analysis of DICER (DCR) and NANOG in protein extracts from DICER conditional mESCs 8 days after treatment with ethanol (WT) or 4-OHT (KO) in three independent biological replicates (BR1–3). ACTIN-β (ACT-β) was used as an internal control.

E Densiometric quantification of the relative difference in DCR levels from (D). Data are represented as mean ± SD, and each point corresponds to the results of one independent biological replicate. Two-tailed paired t-test P-value = 0.010.

F Percentage of proliferating mESCs after 8 days of treatment with ethanol (WT) or 4-OHT (KO) in three independent biological replicates. Data are represented as mean ± SD, and each point corresponds to the results of one independent biological replicate. Two-tailed paired t-test P-value = 0.011.

G Fold change (FC) in *Oct4* (two-tailed t-test P-value = 0.34), *Nanog* (paired two-tailed t-test P-value = 0.084), *Myc* (paired two-tailed t-test ***P-value = 0.008), *Sox2* (paired two-tailed t-test P-value = 0.52) and *Dicer* (paired two-tailed t-test ***P-value = 4×10^{-4}; x-axis) expression in mESCs after 8 days of treatment with 4-OHT (KO) relative to ethanol-treated cells (WT) measured by RT–qPCR for three independent biological replicates (y-axis). Transcript expression in WT and KO cells was normalized by Actin-β and *PolymeraseII* expression. Horizontal dashed line represents a KO/WT fold change in expression of 1. Each point represents the ratio of one independent biological replicate.

H, I Western blot using antibodies against mouse OCT-4 (two-tailed t-test P-value = 0.880) (H) and NANOG (two-tailed t-test P-value = 0.700) (I) in protein extracts from DICER conditional mESCs 8 days after treatment with ethanol (WT) or 4-OHT (KO) for three independent biological replicates (BR1–3, same samples used in panel (D)). ACTIN-β (ACT-β) was used as an internal control and to determine the relative difference in OCT4 and NANOG levels represented in bar plot. Data are represented as mean ± SD, and each point corresponds to the results of one independent biological replicate.

J Expression of miR-295-3p and miR-290-3p relative to sno-202 (x-axis) in *Dcr* conditional mESCs 8 days after treatment with ethanol (WT) or 4-OHT (KO) (y-axis) for three independent biological replicates (Paired two-tailed t-test, *P-value = 0.010 for miR-290-3P and 0.015 for miR-295-3P). Data are represented as mean ± SD, and each point corresponds to the results of one independent biological replicate.

K Fold change (FC) in *Cdkn1a*, *Lats2* and *Rbl2* (x-axis) expression in mESCs after 8 days of treatment with 4-OHT (KO) relative to WT (ethanol-treated) measured by RT–qPCR for three independent biological replicates (y-axis). Transcript expression in WT and KO cells was normalized by Actin-β and *PolymeraseII* expression. Horizontal dashed line represents a KO/WT fold change in expression of 1 (paired two-tailed t-test WT vs KO, **P-value = 0.005 for *Cdkn1a and* 0.002 for *Lats2*). Data are represented as mean ± SD, and each point corresponds to the results of one independent biological replicate. Each point represents the ratio of one independent biological replicate.

Data information: Statistics: NS-P-value > 0.05, *P-value < 0.05, **P-value < 0.01 and ***P-value < 0.001. Uncropped blots used to assemble panels (C, F and G) are provided in Fig EV1 Source Data.

© 2021 The Authors

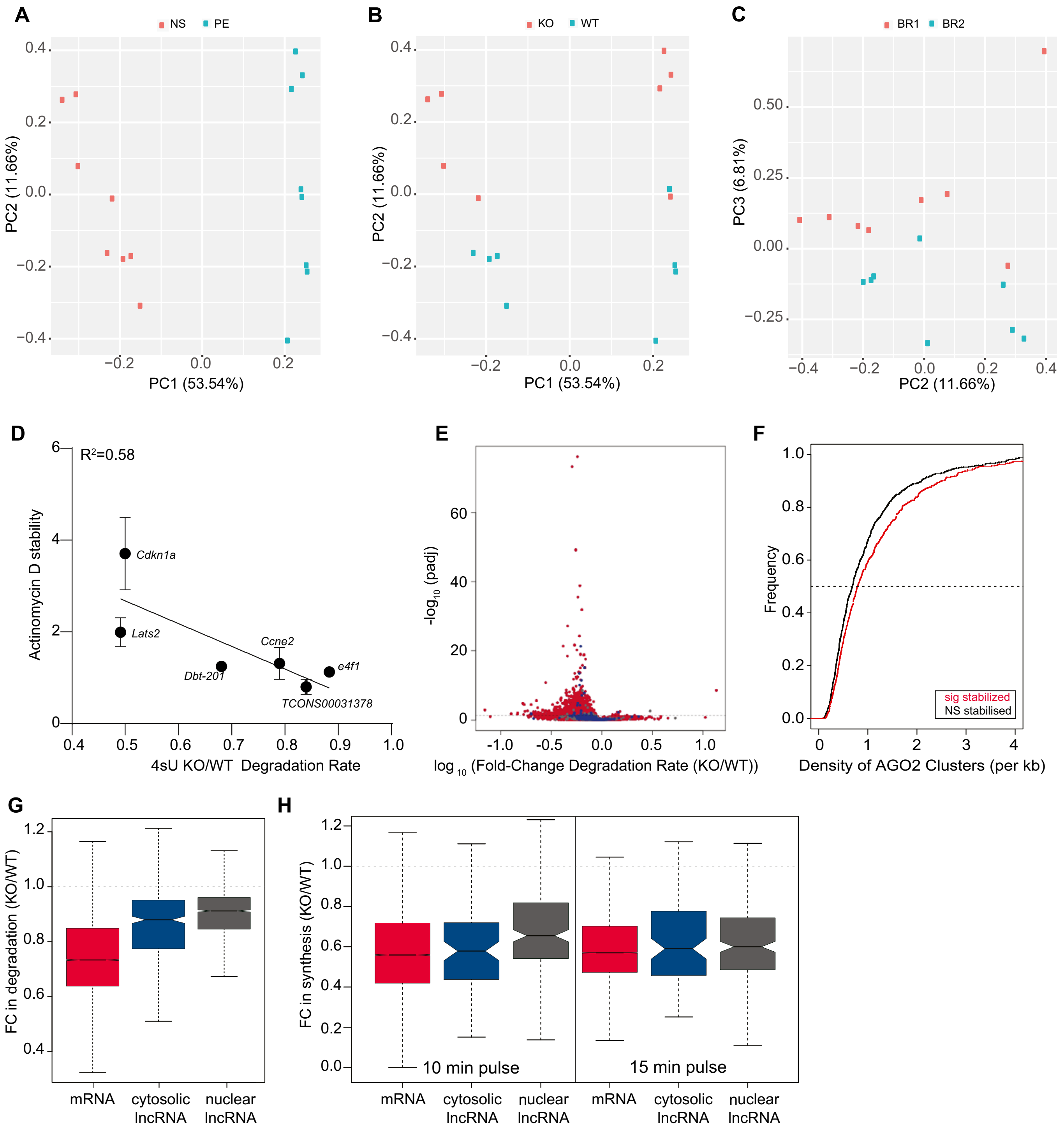

Figure EV2.

 © 2021 The Authors

◀ **Figure EV2. 4sU metabolic labelling in wild-type and miRNA-depleted mESC.**

A–C Principal component analysis of gene expression. The first 2 axes (PC1 and PC2) separate samples into (A) RNA fraction, newly synthesized RNA (NS, red) and pre-existing RNA (PE blue) and (B) cell type, DICER-depleted (KO, red) and wild-type (WT, blue). (C) PC2 and PC3 separate biological replicates (BR1 red and BR2 blue).

D Fold change in 4sU degradation rate between KO and WT cells (*X*-axis) is inversely correlated with the fold change in relative expression between KO and WT after 8 h of treatment with actinomycin-D relative to 0 h actinomycin-D treatment timepoint (*y*-axis). Points represent the mean and standard deviation based on three independent mESC biological replicates.

E Volcano plot showing the adjusted *P*-value (*y*-axis) as a function of the fold change in degradation rate, estimates based on the 15 min pulse, between KO and WT cells (*x*-axis) for protein-coding genes (red), cytosolic (blue) and nuclear (grey) lncRNAs. Each point represents a transcript and the horizontal dashed line represents the significance cut-off.

F Cumulative distribution plot of the density of AGO2 clusters in the 3'untranslated regions of AGO-2 bound mRNAs (AGO2 cluster > 0) whose degradation rates were either significantly (red) or not significantly changed (black) between KO and WT cells, based on the 15 min pulse estimates. Density of clusters presented in this analysis was estimated based on data from (Leung *et al*, 2011).

G Distribution of the fold change (FC) in degradation rate of mRNAs (red), cytosolic (blue) and nuclear (grey) lncRNAs in 4-OHT-treated (KO) relative to ethanol-treated (WT) cells after 8 days of treatment (estimated based on the 15 min 4sU pulse), horizontal dashed line represents a KO/WT FC in degradation rate of 1. Central band of boxplot represents median, box depicts 25–75 quantiles of distribution, and whiskers represent the 5th and 95th quantiles of the distribution (lower and upper whiskers, respectively). Rate inference was performed based on the results from two independent biological replicates.

H Distribution of the fold change (FC) in synthesis rate of mRNAs (red), cytosolic (blue) and nuclear (grey) lncRNAs, in 4-OHT-treated (KO) relative to ethanol-treated (WT) cells after 8 days of treatment. Results for the 10- and 15-min pulse are presented separately, horizontal dashed line represents a KO/WT FC in synthesis rate of 1. Central band of boxplot represents median, box depicts 25–75 quantiles of distribution, and whiskers represent the 5th and 95th quantiles of the distribution. Rate inference for each labelling duration timepoint was performed based on two independent biological replicates.

© 2021 The Authors

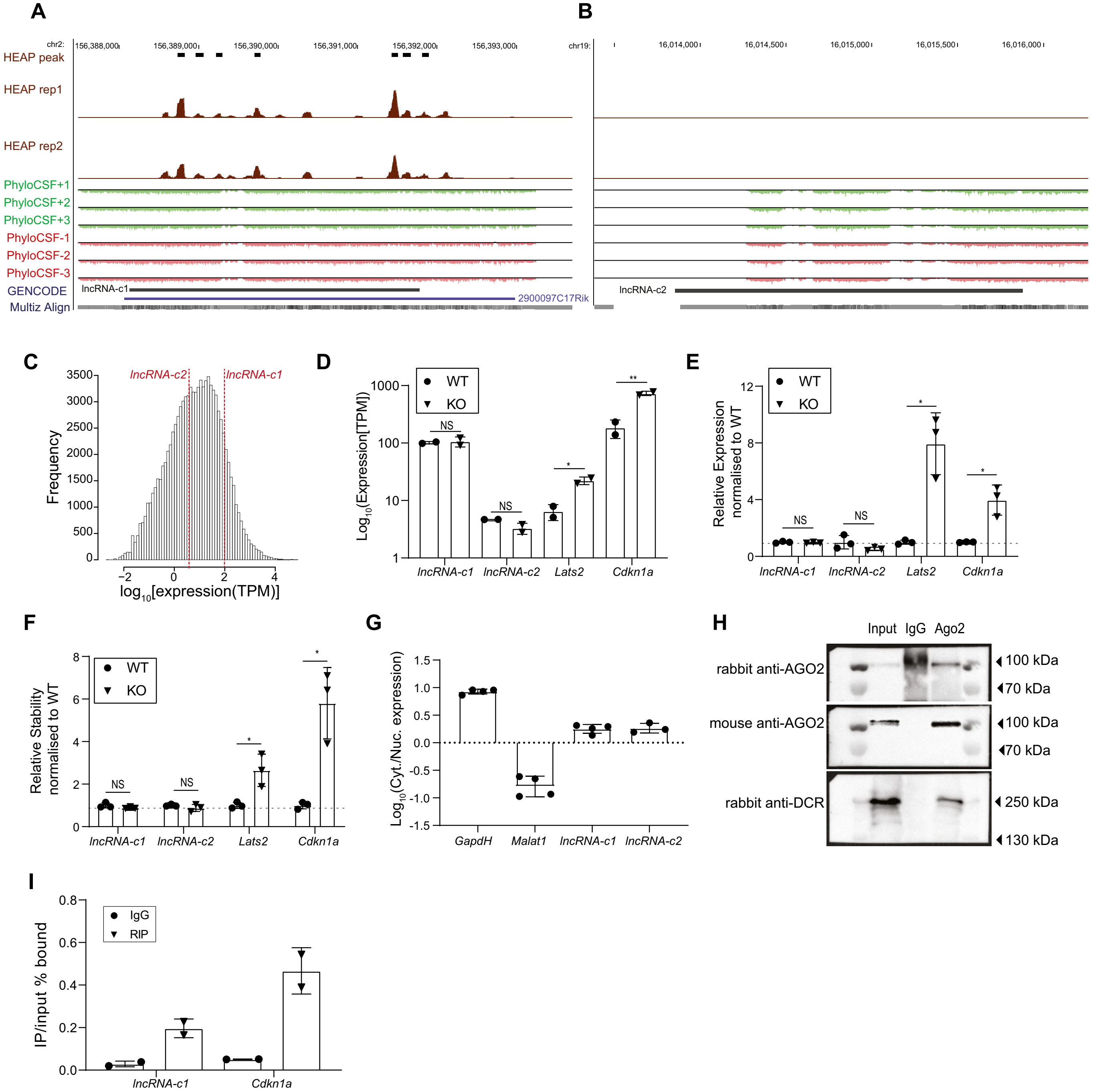

Figure EV3.

 © 2021 The Authors

◀ **Figure EV3. Selection of lncRNA candidates to test prerequisite of target translation for miRNA-dependent target destabilization.**

A, B Genome browser view of the region encompassing *lncRNA-c1* (A) or *lncRNA-c2* (B) Halo-enhanced AGO2 pull-down peaks (Li *et al*, 2020) and read density for two independent replicates (between 0–127) as well as PhyloCSF scores (between −15 and 15) in all possible reading frames are depicted. Gencode annotated genes are annotated in blue, and the candidates are annotated in black.

C Distribution of gene expression ($\log_{10}$(TPM), *x*-axis) for all mESC-expressed transcripts. Red dotted horizontal line indicates the expression of *lncRNA-c1* and *lncRNA-c2*.

D Expression of *lncRNA-c1*, *lncRNA-c2*, *Lats2* and *Cdkn1a* ($\log_{10}$TPM), measured by RNA sequencing, in two independent biological replicates of 4-OHT-treated (KO, triangles) and wild-type (WT, circles) mESCs after 8 days of treatment. Each point represents the expression measured in one biological replicate. Data are represented as mean ± SD, and each point corresponds to the results of one independent biological replicate.

E Expression of *lncRNA-c1*, *lncRNA-c2*, *Lats2* and *Cdkn1a* normalized to ethanol-treated cells (WT, circles), as measured by RT–qPCR in three independent WT and 4-OHT-treated (KO, triangles) mESCs biological replicates following 8 days of treatment. Transcript expression was normalized by expression of *Actin-β* and *PolymeraseII*. Each point represents the normalized expression measured in one biological replicate, horizontal dashed line represents WT-normalized expression of 1. Data are represented as mean ± SD, and each point corresponds to the results of one independent biological replicate.

F Stability measured as the relative amount of transcript detected after 8 h of transcription block using actinomycin-D, for *lncRNA-c1*, *lncRNA-c2*, *Lats2* and *Cdkn1a* expression in 4-OHT-treated (KO, triangles) cells normalized to ethanol-treated (WT, circles) cells following 8 days of treatment. Expression was measured by RT–qPCR in three independent biological replicates of mESCs. Transcript expression was normalized by expression of *Actin-β* and *PolymeraseII*. Each point represents the normalized expression measured in one biological replicate, horizontal dashed line represents WT-normalized stability of 1. Data are represented as mean ± SD, and each point corresponds to the results of one independent biological replicate.

G Log_{10} of the fold change in absolute expression in the cytosolic fraction relative to the nuclear fraction ((Cyt./Nuc. Expression), *y*-axis), as measured by RT–qPCR, for *lncRNA-c1*, *lncRNA-c2*, nuclear (*Malat1*) and cytosolic (*Gapdh*) control transcripts. RT–qPCR analyses of four independent biological replicates of mESC cytosolic fractionation experiments were tested for *GapdH*, *Malat1* and *lncRNA-c1*. Three independent mESC biological replicates were tested for *lncRNA-c2*. Each point represents the Log_{10} ratio of expression measured in one independent biological replicate. Data are represented as mean ± SD, and each point corresponds to the results of one independent biological replicate.

H Representative Western blot analysis of protein extracts from input, AGO2-RIP and IgG control. AGO2 was probed with rabbit AGO2 antibody (top panel). After membrane stripping and re-probing with mouse anti-AGO2 (middle panel) unspecific band in IgG was cleared. Probing with rabbit antibody confirmed the presence of DICER specifically in the input and AGO2-RIP samples (lower panel).

I RT–qPCR quantification of *lncRNA-c1* and *Cdkn1a* (*x*-axis) bound in AGO2-IP (triangles) relative to input and unspecific IgG (circles) antibody relative to input (*y*-axis) in two independent mESC biological replicates. Each point represents the IgG/INPUT % ratio measured in one biological replicate. Data are represented as mean ± SD, and each point corresponds to the results of one independent biological replicate. Data information: Statistics: NS-*P*-value > 0.05, **P*-value < 0.05 and ***P*-value < 0.01.

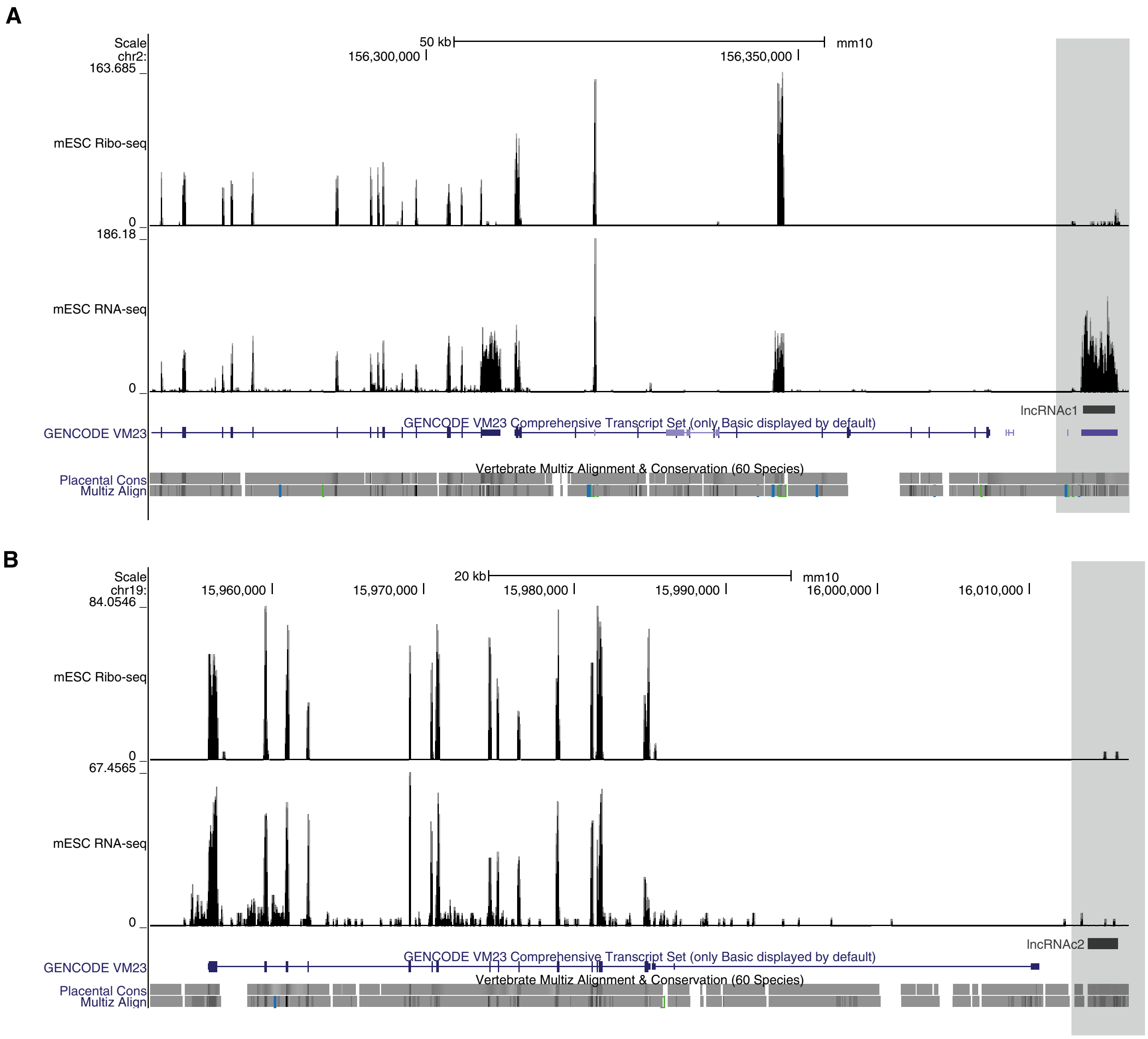

Figure EV4. Candidate lncRNAs are only weakly associated with ribosomes.

A, B Genome browser view of the region encompassing (A) *lncRNA-c1* or (B) lncRNA-c2. The density of ribosomal profiling (mESC Ribo-seq, top) and RNA sequencing reads (mESC RNA-Seq, bottom) is represented in the (*y*-axis). GENCODE annotated genes in the depicted regions are annotated in blue, and the candidate annotations are highlighted in grey.

© 2021 The Authors

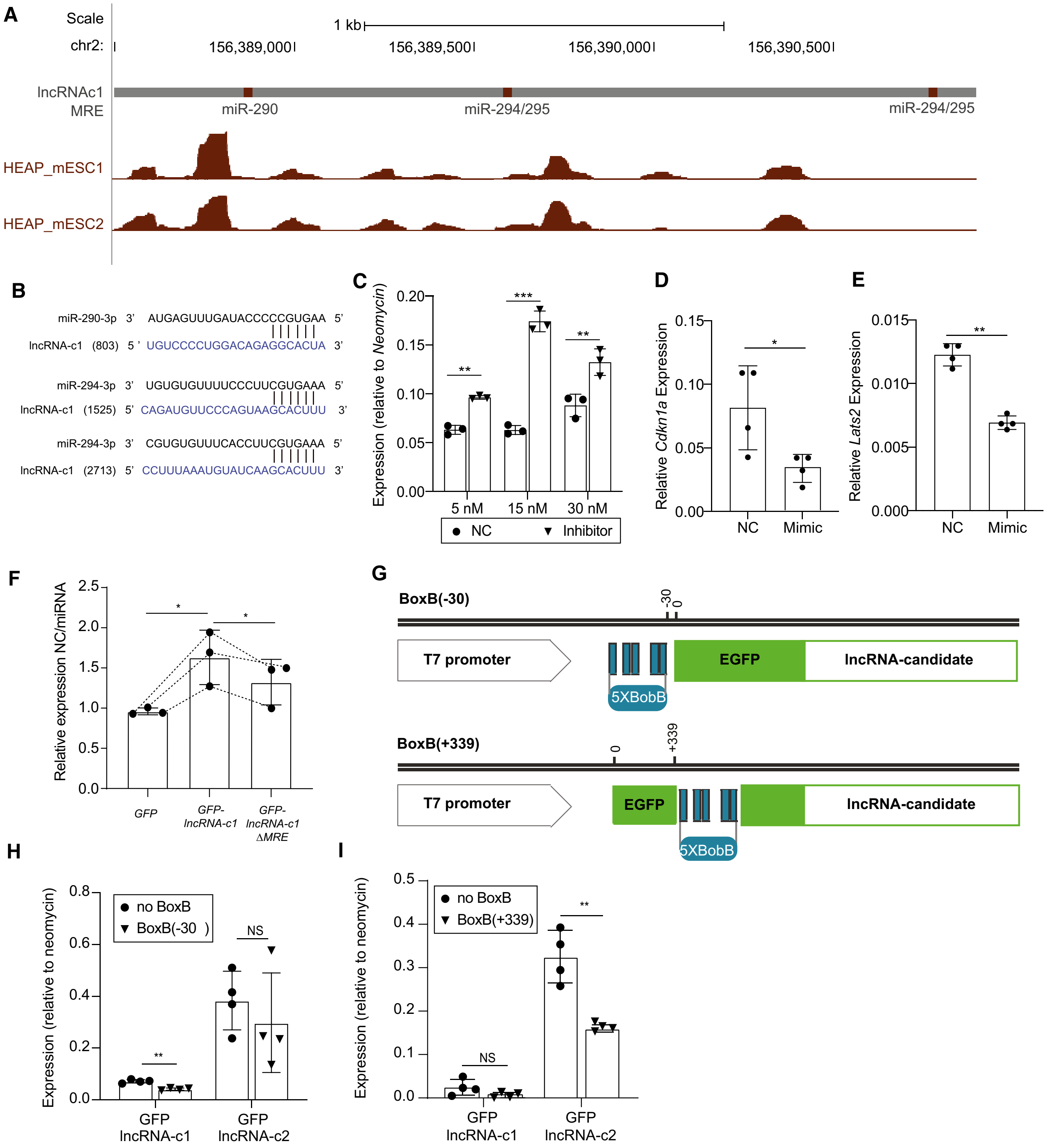
A
Scale
chr2:
1 kb
156,389,000
156,389,500
156,390,000
156,390,500
lncRNAc1
MRE
miR-290
miR-294/295
miR-294/295
HEAP_mESC1
HEAP_mESC2
B
miR-290-3p 3' AUGAGUUUGAUACCCCCGUGAA 5'
lncRNA-c1 (803) 5' UGUCCCCUGGACAGAGGCACUA 3'
miR-294-3p 3' UGUGUGUUUUCCCUUCGUGAAA 5'
lncRNA-c1 (1525) 5' CAGAUGUUCCCAGUAAGCACUUU 3'
miR-294-3p 3' CGUGUGUUUCACCUUCGUGAAA 5'
lncRNA-c1 (2713) 5' CCUUUAAAUGUAUCAAGCACUUU 3'
C
Expression (relative to Neomycin)
5 nM
15 nM
30 nM
NC
Inhibitor
D
Relative Cdkn1a Expression
NC
Mimic
E
Relative Lats2 Expression
NC
Mimic
F
Relative expression NC/miRNA
GFP
GFP-lncRNA-c1
GFP-lncRNA-c1 ΔMRE
G
BoxB(-30)
T7 promoter
5XBobB
EGFP
lncRNA-candidate
BoxB(+339)
H
Expression (relative to neomycin)
no BoxB
BoxB(-30)
NS
GFP lncRNA-c1
GFP lncRNA-c2
I
Expression (relative to neomycin)
no BoxB
BoxB(+339)
NS

Figure EV5.

◀ **Figure EV5. Relieving miRNA binding or translation levels of *GFP-lncRNA-c1* reduces its susceptibility to miRNA-dependent transcript destabilization.**

A Genome browser view of the region encompassing miR-290 and miR-295/4 MREs (red) within *lncRNA-c1*. Halo-enhanced AGO2 pull-down (Li *et al*, 2020) read density for two independent replicates (between 0–127 is represented in the *y*-axis).

B Pairwise alignment between miR-290-3p (top) and miR-294-3p (bottom) and respective predicted miRNA response elements (MRE) within *lncRNA-c1*. Seed-complementary MRE start position within annotated *lncRNA-c1* transcript is indicated inside parentheses.

C *GFP-lncRNA-c1* expression 24 h following transfection of mESCs with 5, 15 or 30 mM mmu-miR294-3p inhibitors (Inhibitor, triangles) or small RNA negative control (NC, squares). Each point corresponds to the results of one technical replicate. Transcript expression was first normalized by the amount of *Actin-β* and *GapdH* and next by the total amount of transfected vectors per cell estimated based on levels of relative *Neomycin* expression. Comparison between the expression in cells transfected with 5, 15 and 30 nM of Negative control or inhibitor unpaired two-tailed *P*-value = 0.006, 0.001 and 0.003, respectively. Data are represented as mean ± SD, and each point corresponds to the results of one independent biological replicate.

D, E Relative *Cdkn1a* (D) and *Lats2* (E) expression following transfection of mmu-miR-294-3p and mmu-miR-295-3p equimolar mixes (Mimic) or negative control small RNA (NC). Each point corresponds to the results of one independent biological replicate. Transcript expression was first normalized by the amount of *Actin-β* and *PolymeraseII*. Data are represented as mean ± SD, and each point corresponds to the results of one independent biological replicate.

F Fold change in expression(*y*-axis) of *GFP*, *GFP-lncRNA-c1* and *GFP-lncRNA-c1-MREΔ* (*x*-axis) in miRNA-depleted cells transfected with negative control (NC) relative to miRNA-depleted cells transfected with miRNA mimics (miRNA) (*y*-axis). Each point corresponds to the results of one independent biological replicate. Transcript expression was first normalized by the amount of *Actin-β* and *PolymeraseII* and next by the total amount of transfected vectors per cell estimated based on levels of relative *Neomycin* expression. Two-tailed paired *t*-test *P*-value = 0.043. Data are represented as mean ± SD, and each point corresponds to the results of one independent biological replicate.

G Schematics of the BoxB insertion in GFP-lncRNA-candidate constructs.

H Relative expression of GFP in mESCs (*y*-axis) transfected with *GFP-lncRNA-c1, GFP-lncRNA-c2* (noBoxB, circles) and *BoxB(−30)-GFP-lncRNA-c1, BoxB(−30)-GFP-lncRNA-c2* (BoxB(−30), triangles; *x*-axis). Four independent mESC biological replicates were transfected and analysed by RT–qPCR. Transcript expression was first normalized by the amount of *Actin-β* and *PolymeraseII* and next by the total amount of transfected vectors per cell estimated based on levels of relative *Neomycin* expression. Each point corresponds to the results of one independent biological replicate. Comparison of the relative expression in mESC of constructs with or without BoxB(−30) paired two-tailed *t*-test *P*-value = 0.004 and 0.200 for *GFP-lncRNA-c1* and *GFP-lncRNA-c2*, respectively. Data are represented as mean ± SD, and each point corresponds to the results of one independent biological replicate.

I Relative expression of GFP in mESCs (*y*-axis) transfected with *GFP-lncRNA-c1, GFP-lncRNA-c2* (noBoxB, circles) and *BoxB(+339)-GFP-lncRNA-c1, BoxB(+339)-GFP-lncRNA-c2* (BoxB(+339), triangles; *x*-axis). Four independent mESC biological replicates were analysed. Transcript expression was first normalized by the amount of *Actin-β* and *PolymeraseII* and next by the total amount of transfected vectors per cell estimated based on levels of relative *Neomycin* expression. Each point corresponds to the results of one independent biological replicate. Comparison of the relative expression in mESC of constructs with or without BoxB(+339) paired two-tailed *t*-test *P*-value = 0.21 and 0.010 for *GFP-lncRNA-c1* and *GFP-lncRNA-c2*, respectively). Data are represented as mean ± SD, and each point corresponds to the results of one independent biological replicate.

Data information: Statistics: NS-*P* > 0.05, **P* < 0.05, ***P* < 0.01 and ****P* < 0.001 two-tailed paired *t*-test.

 © 2021 The Authors
