# Supplementary material for: Translation is required for miRNA‐dependent decay of endogenous transcripts
Source: EMBO J. 2020 Dec 10;40(3):e104569. doi: 10.15252/embj.2020104569 (PMC7849302; doi:10.15252/embj.2020104569)
Supplement: Supplementary file 5 — Source Data for Expanded View [file EMBJ-40-e104569-s006.zip › EMBOJ-2020-104569R1-Figure_EV1_Source_Data-sd.pdf]

A.

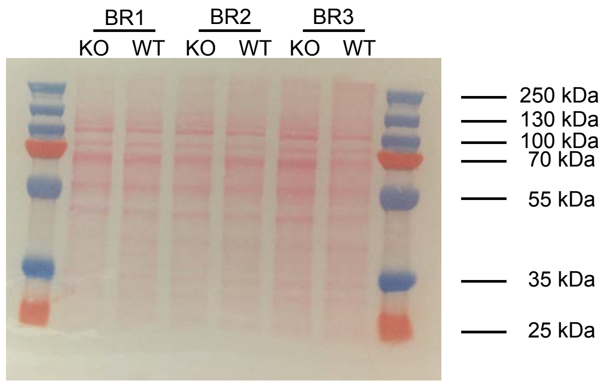

B.

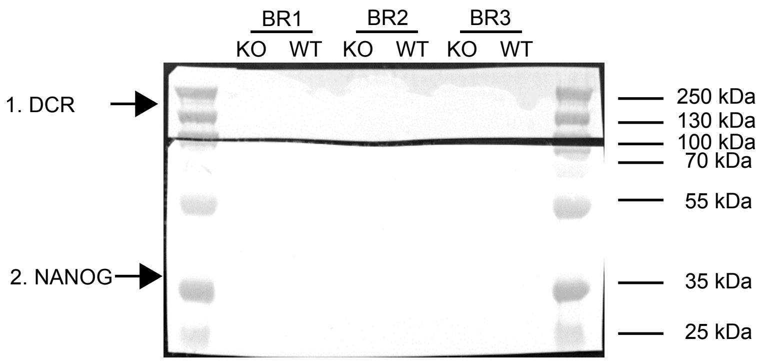

Uncropped blot

C.

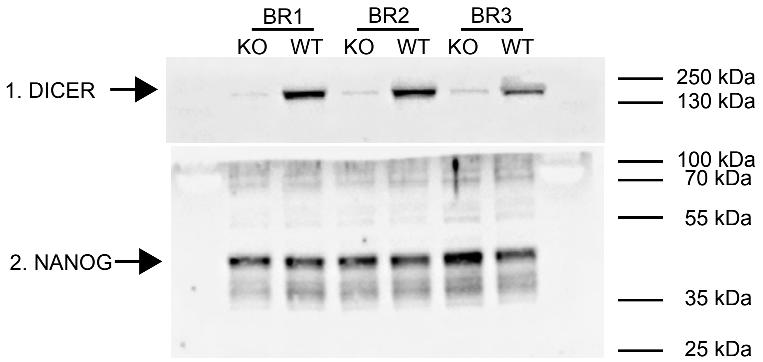

D.

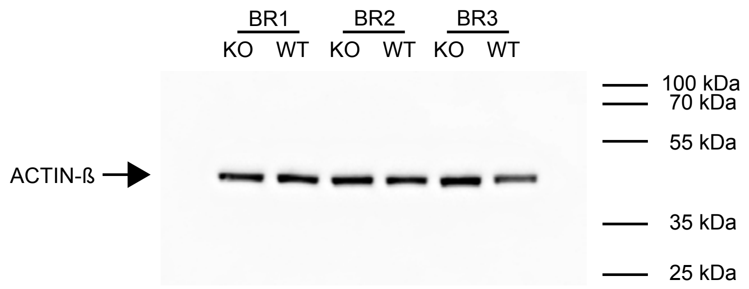

Source data EV1D - Uncropped blots for NANOG and DICER detection (A) Ponceau S Solution staining of gel-separated, nitrocellulose membrane-transferred proteins from 3 Biological Replicates (BR) of 4-OHT treated (KO) and ethanol treated (WT) mESC after 8 days of treatment. (B) Non-luminescent Image of cut Membrane portions used to simultaneously detect for DCR (1) and NANOG (2). (C) Simultaneous chemiluminescent detection of HRP-bound substrates on portions 1 and 2 of the membrane. (D) Chemiluminescent detection of HRP-coupled substrates following Sodium-Azide dependent deactivation of HRP-coupled antibodies and re-probing for ACTIN-β. Size in kiloDaltons (kDa) is represented on left side of images.

A.

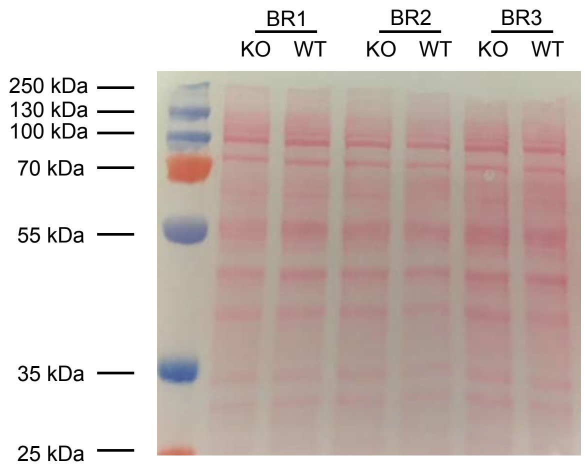

B.

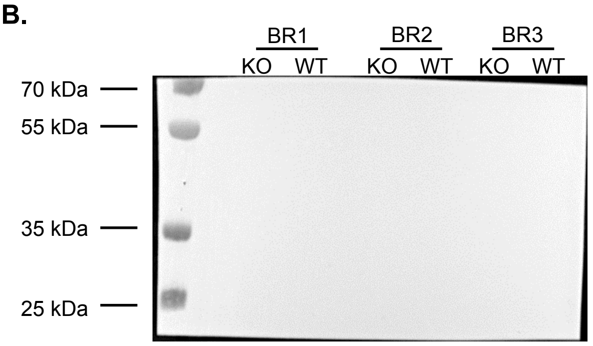

C.

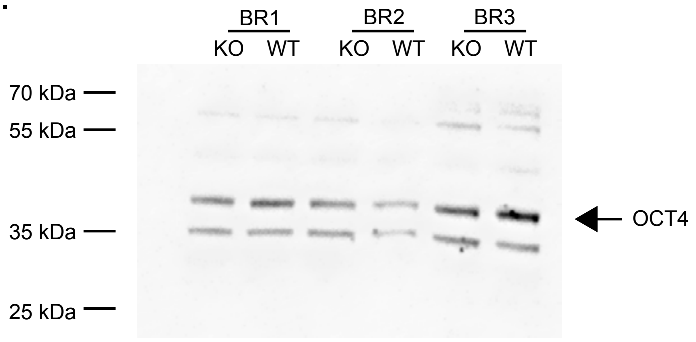

D.

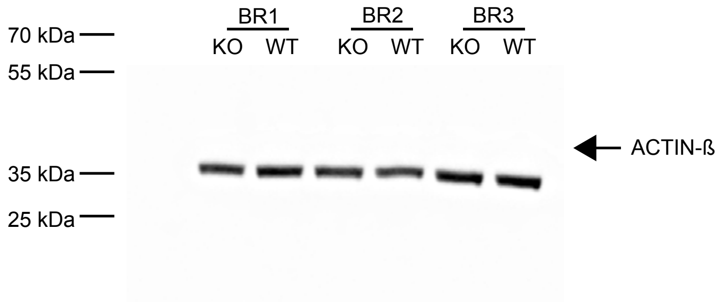

Source data EV1H - Uncropped blots for OCT4 detection. (A) Ponceau S Solution staining of gel-separated, nitrocellulose membrane-transferred proteins from 3 Biological Replicates (BR) of 4-OHT treated (KO) and ethanol treated (WT) mESC after 8 days of treatment. (B) Non-luminescent Image of membrane following probing for OCT-4. (C) Chemiluminescent detection of HRP-bound substrates on membrane following probing for OCT-4. (D) Chemiluminescent detection of HRP-coupled substrates following Sodium-Azide dependent deactivation of HRP-coupled antibodies and re-probing for ACTIN-β. Size in kiloDaltons (kDa) is represented on left side of images.
