# Supplementary material for: Translation is required for miRNA‐dependent decay of endogenous transcripts
Source: EMBO J. 2020 Dec 10;40(3):e104569. doi: 10.15252/embj.2020104569 (PMC7849302; doi:10.15252/embj.2020104569)
Supplement: Supplementary file 7 — Source Data for Figure 4 [file EMBJ-40-e104569-s005.pdf]

A.

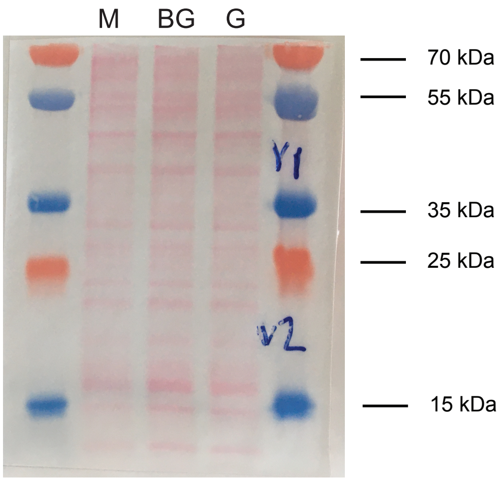

B.

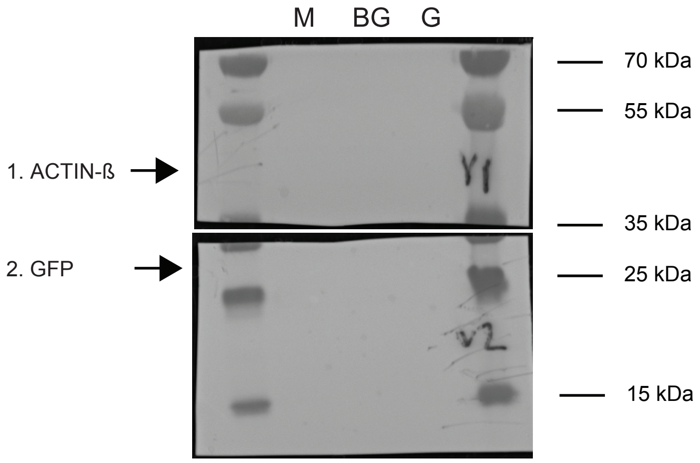

C.

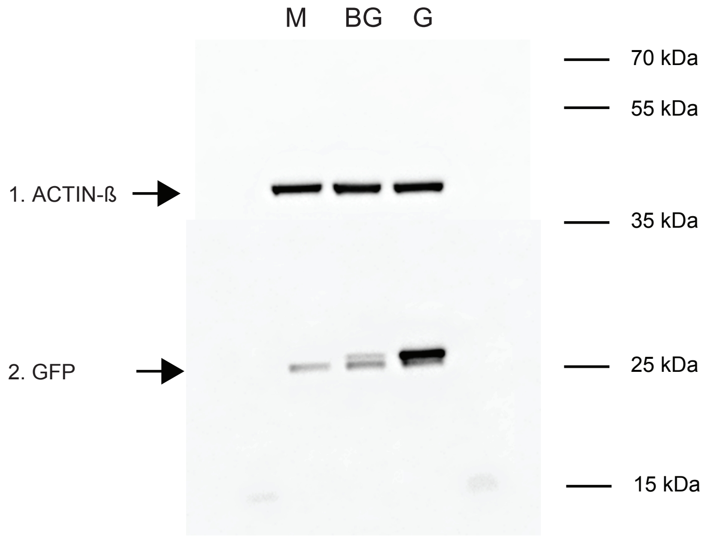

Source data Figure 4F - Uncropped blots for BoxB(-30)GFP detection following transfection in wildtype (WT) mESCs. (A) Ponceau S Solution staining of gel-separated, nitrocellulose membrane-transferred proteins from mESCs transfected with mock (M), BoxB(-30)GFP (BG) and GFP (G). (B) Non-luminescent Image of membrane portions following simultaneous probing for ACTIN-β (1) and GFP (2). (C) Chemiluminescent detection of HRP-bound substrates on membrane portions following simultaneous probing and detection for ACTIN-β (1) and GFP (2).

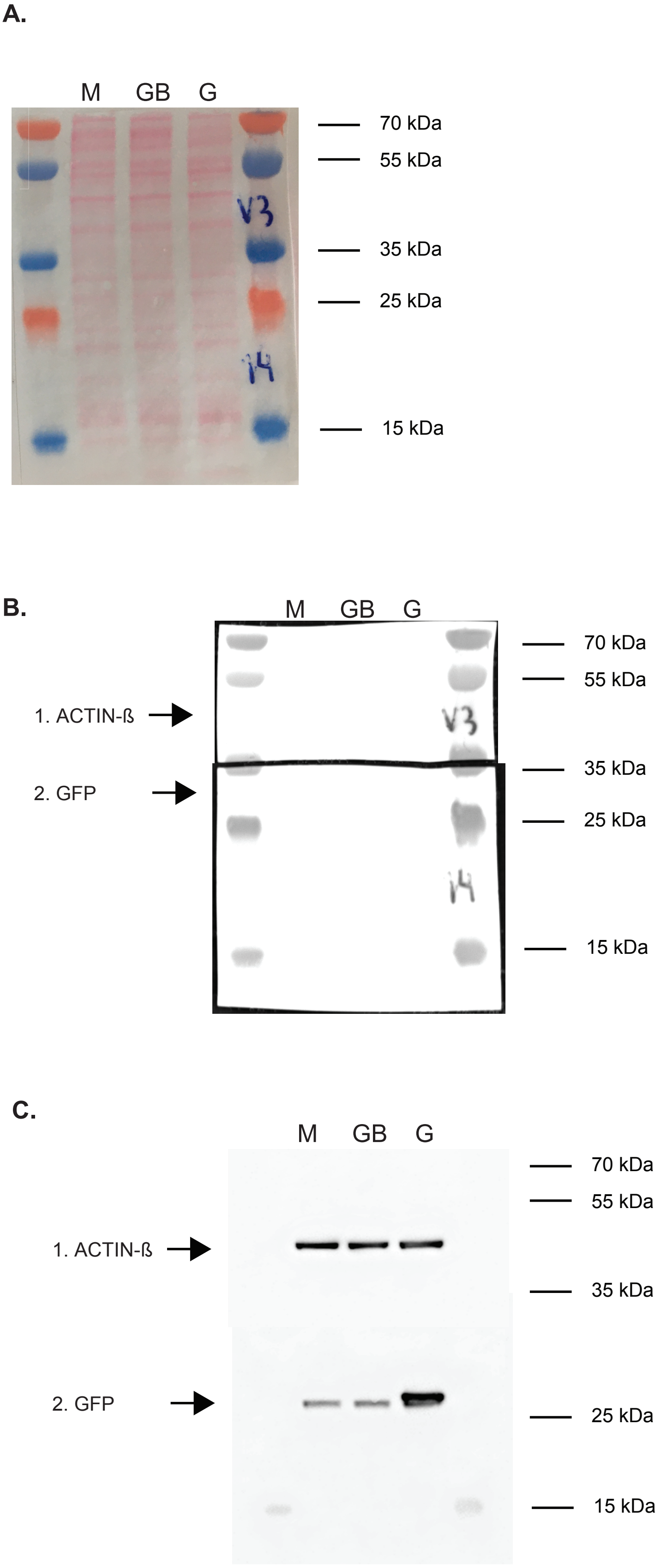

Source data Figure 4H - Uncropped blots for BoxB(+339)GFP detection following transfection in wildtype (WT) mESCs. (A) Ponceau S Solution staining of gel-separated, nitrocellulose membrane-transferred proteins from mESCs transfected with mock (M), BoxB(+339)GFP (GB) and GFP (G). (B) Non-luminescent Image of membrane portions following simultaneous probing for ACTIN-β (1) and GFP (2). (C) Chemiluminescent detection of HRP-bound substrates on membrane portions following simultaneous probing and detection for ACTIN-β (1) and GFP (2).
